# Glucose metabolism in systemic juvenile idiopathic arthritis

**DOI:** 10.1186/s12969-022-00714-6

**Published:** 2022-07-29

**Authors:** Papatsorn Suppasit, Soamarat Vilaiyuk, Preamrudee Poomthavorn, Sarunyu Pongratanakul, Patcharin Khlairit, Pat Mahachoklertwattana

**Affiliations:** Department of Pediatrics, Faculty of Medicine Ramathibodi Hospital, Mahidol University, 270 Rama VI Road, Bangkok, 10400 Thailand

**Keywords:** Insulin sensitivity, Oral glucose tolerance test, Insulin resistance, Prediabetes, Diabetes

## Abstract

**Background:**

Systemic juvenile idiopathic arthritis (SJIA) is a chronic systemic inflammatory disease in children. Overproduction of inflammatory cytokines in SJIA resembles that in adult onset Still disease. Chronic inflammation causes insulin resistance and consequently leading to abnormal glucose metabolism. Adults with rheumatoid arthritis (RA) have increased risks of abnormal glucose metabolism and diabetes. To date, glucose metabolism in patients with SJIA has not been elucidated.

**Methods:**

Patients with SJIA aged 4–25 years were recruited. All patients underwent an oral glucose tolerance test (OGTT). Indices of insulin sensitivity [homeostasis model assessment for insulin resistance (HOMA-IR) and whole-body insulin sensitivity index (WBISI)] and β-cell function [insulinogenic index (IGI) and disposition index (DI)] were calculated. Obese children with normoglycemia who underwent the OGTT were served as a control group.

**Results:**

A total of 39 patients with SJIA, aged 4–25 years, median (IQR) BMI SDS was 0.1 (-0.5 to 1.7). Patients were divided into 2 groups, overweight/obese (OW/OB) (*n* = 11) and lean (*n* = 28). Only one obese patient had prediabetes and none had diabetes. In comparison with sex- and age-matched OW/OB controls (*n* = 33), OW/OB patients with SJIA had higher insulin resistance [median (IQR) HOMA-IR: 2.6 (2.1–3.3) *vs* 1.5 (0.8–2.0), *p* = *0.001*], lower insulin sensitivity [median (IQR) WBISI: 3.7 (2.7–5.9) *vs* 5.4 (4.5–8.7), *p* = *0.024*], and higher insulin secretion [median (IQR) IGI: 2.5 (2.0–3.5) *vs* 1.0 (0.8–1.9), *p* = *0.001*]**.** In lean patients with SJIA, insulin sensitivity indices seemed to be comparable with those of lean controls.

**Conclusions:**

Overweight/obese children with SJIA seemed to have increased insulin resistance and thus may have an increased risk for developing diabetes.

## Introduction

Systemic juvenile idiopathic arthritis (SJIA) is a category of juvenile idiopathic arthritis (JIA) according to the International League of Associations for Rheumatology classification criteria [1]. It is a chronic systemic inflammatory disease, leading to disability in children. Our previous study demonstrated that the most common JIA category was SJIA (34%), followed by enthesitis-related arthritis (ERA) (25%) [2]. Previous studies revealed that SJIA was the most prevalent subtype of JIA in Southeast Asia, followed by enthesitis-related arthritis (ERA), whereas oligoarthritis was the most common form in western countries [3].

Pathogenesis mechanisms of SJIA were dysregulation of immune system, and overproduction of inflammatory cytokines [tumor necrosis factor-α (TNF-α), interleukin-1 (IL-1) and interleukin-6 (IL-6)] resembled those found in adult rheumatoid arthritis (RA) [4]. RA in adults are associated with increased risks of diabetes and metabolic syndrome (MS). Previous studies have shown that inflammatory cytokines can induce islet β-cell apoptosis, insulin resistance and consequently leading to hyperglycemia [5, 6]. Medications for SJIA treatment include nonsteroidal anti-inflammatory drugs (NSAIDs), glucocorticoids, disease-modifying antirheumatic drugs (DMARD) such as methotrexate (MTX) and biologic agents. Since glucocorticoids exert multiple effects on glucose metabolism, long-term glucocorticoid treatment could increase risks of diabetes. Glucocorticoid-induced hyperglycemia is improved with dose reduction and could be reversed with glucocorticoid discontinuation [7]. Two major factors, chronic inflammation and long-term glucocorticoid therapy, cause insulin resistance in SJIA and consequently leads to prediabetes and diabetes during childhood period. However, to date, no recommendation for glucose metabolism screening in these patients is available.

Obesity and overnutrition induce state of low-grade inflammation as a result of an accumulation of elevated levels of glucose and/or lipids in blood stream [8]. In addition, insulin resistance is directly linked to chronic inflammation and oxidative stress [9]. Prediabetes is a condition that blood glucose levels are higher than normal and is associated with an increased risk of type 2 diabetes (T2DM) [10]. Obesity and insulin resistance are the two major factors associated with pathogenesis of T2DM. Therefore, SJIA, a model of chronic inflammation in children, may be associated with an increased risk of abnormal glucose metabolism. To our knowledge, glucose metabolism in patients with SJIA has not been reported. The purpose of this study was to evaluate glucose metabolism in children with SJIA.

## Methods

### Study design

We conducted a cross-sectional study in patients with SJIA who were followed up in Pediatric Rheumatology Clinic at Ramathibodi Hospital, Bangkok, Thailand between 2019 and 2021. The diagnosis of SJIA required the presence of arthritis of unknown etiology that began before the age of 16 years and persisted for at least 6 weeks preceded by quotidian fever at least 2 weeks, plus 1 or more of the following findings: typical evanescent, non-fixed erythematous rash, hepatomegaly or splenomegaly, generalized lymphadenopathy and serositis [1]. Exclusion criteria were patients with acute conditions (trauma, severe infection, exacerbation of chronic disease) and patients who were diagnosed with type 1 and type 2 diabetes prior to the study enrollment. None of the SJIA patients in our Rheumatology Clinic has had documented diabetes. Oral glucose tolerance test (OGTT) was performed in all SJIA patients. The control group is a cohort of children with simple obesity who underwent the routine OGTT in our Pediatric Endocrine Clinic according to their clinical criteria. Patients and their parents provided written informed consents before participation. This study was approved by the Ethics Committee of the Faculty of Medicine Ramathibodi Hospital, Mahidol University (MURA 2020/599). Demographic characteristics were recorded for each patient at diagnosis of SJIA: age, sex, pubertal status, disease duration, previous and concomitant therapies. Juvenile Arthritis Disease Activity Score (JADAS-27) and Systemic Manifestation Score (SMS) were used for assessing disease activity. [11, 12]. JADAS-27 was derived from 4 variables obtained on the same day of the OGTT testing. These 4 variables comprise the total number of active joints (27 joints were evaluated), erythrocyte sedimentation rate (ESR) normalized to a 0–10 scale ([ESR mm/h-20]/10), the physician's global assessment score (0–10) and the parent's or patient's global assessment of overall well-being score (0–10). Minimum and maximum JADAS-27 scores are 0 and 57, respectively [11]. In this study, the cumulative JADAS-27 scores across the course of the patients’ disease were estimated by summing the scores obtained serially since the diagnosis until the enrollment. The systemic manifestation score (SMS) was created by considering extraarticular symptoms as follows: fever = 1 point for temperature (T) of 37–38 °C, 2 points for T 38–39 °C, 3 points for T 39–40 °C, 4 points for T > 40 °C; rash = 1 point; generalized lymphadenopathy = 1 point; hepatomegaly and/or splenomegaly = 1 point; serositis = 1 point; anemia (hemoglobin < 9 g/dl) = 1 point; platelet count >  × 10^9^/L or ferritin > 500 ng/mL = 1 point [12]. Patients were divided into 3 groups according to disease status: 1) patient with systemic features with or without arthritis, 2) patient with arthritis but no systemic features and 3) patient with inactive disease [13]. Pubertal maturation was assessed using the Tanner stage [14, 15]. Body mass index (BMI) was calculated using the formula: weight (kg)/height^2^ (m^2^). Owing to the wide age range of the enrolled patients, for comparison, BMI standard deviation score (BMI SDS) also presented. BMI SDS was calculated from WHO standards [16, 17]. Overweight and obesity were defined as BMI SDS of 1–1.99 and 2 or greater, respectively [16].

### Oral glucose tolerance test (OGTT)

Patients and controls underwent a standard protocol of 2-h OGTT with 1.75 g glucose/kg (max. 75 g) at the Pediatric Endocrine Clinic. After an 8-h fasting, venous blood samplings were obtained for measuring plasma glucose, serum insulin and lipid profiles. Plasma glucose and insulin levels were measured at 30, 60, 90 and 120 min after drinking glucose solution. The diagnoses of diabetes and prediabetes were based on American Diabetes Association (ADA) criteria. Normal fasting glucose was defined as fasting plasma glucose (FPG) < 100 mg/dL. Normal glucose tolerance was defined as 2-h post load glucose of < 140 mg/dL. Impaired fasting glucose (IFG) was defined as fasting plasma glucose (FPG) 100–125 mg/dL. Impaired glucose tolerance (IGT) was defined as 2-h post load glucose between 140 and 199 mg/dL. Diabetes was defined as either FPG 126 mg/dL or greater or 2-h post load glucose 200 mg/dL or greater. Prediabetes was defined as either IFG or IGT or both [18].

### Biochemical analysis

#### Insulin sensitivity and β-cell function assessment

To assess insulin sensitivity, homeostatic model assessment of insulin resistance (HOMA-IR) and whole-body insulin sensitivity index (WBISI) were used. HOMA-IR serves as a hepatic insulin resistance index and WBISI represents peripheral insulin resistance. The higher WBISI indicates more insulin sensitivity. In contrast, the higher HOMA-IR indicates more insulin resistance.

$$HOMA-IR\, = \,fasting\,insulin\,(\mu IU/ml)\, \times \,fasting\,glucose\,(ml/dL)/405$$

$$WBISI\, = \,10000/\,\sqrt {fasting\,insulin\,\,(\mu IU/mL)\, \times \,fasting\,glucose\,(mg/dL)\; \times \,mean\;{\text{glucose}}\,({\text{mg}}/{\text{dL}})\,{\text{x mean insulin}}\, (\mu {\text{IU}}/{\text{mL}})}$$

To assess β-cell function or insulin secretion, insulinogenic index (IGI), disposition index (DI) and ratio of area under the curve (AUC) of insulin to AUC of glucose (AUC ins/AUC glu) were used [19, 20]. The higher IGI, DI and AUC ins/AUC glu indicate greater insulin secretory response to glucose loading.

$$\mathbf{I}\mathbf{G}\mathbf{I} =\mathrm{ \Delta\, insulin\, level\, between\, }0\mathrm\,{ and }\,30\,\mathrm{ min\, }(\mathrm{\mu IU}/\mathrm{mL})/\mathrm{ \Delta\, glucose\, between\, }0\mathrm\,{ and\, }30\mathrm\,{ min }\,(\mathrm{mg}/\mathrm{dL})$$

$$\mathbf{D}\mathbf{I}=\mathbf{W}\mathbf{B}\mathbf{I}\mathbf{S}\mathbf{I}\mathbf{x}\mathbf{I}\mathbf{G}\mathbf{I}$$

**AUC ins/AUC glu** was calculated from AUC of serum glucose and insulin levels obtained during the OGTT using trapezoidal method.

### Metabolic syndrome (MS)

Diagnosis of MS was based on International Diabetes Federation (IDF) criteria which included visceral fat obesity (waist circumference > 90th percentile) plus any two of the other four factors: triglyceride (TG) > 150 mg/dL, high density lipoprotein-cholesterol (HDL-C) < 40 mg/dL, blood pressure > 95th percentile, and either IFG or IGT [21–23]. Due to lack of Thai National Standard for waist circumference, waist circumference percentile according to the previously published reference of Chinese children was used [24].

Data were reported as median and interquartile range (IQR) or mean and standard deviation (SD) for continuous variables as appropriate and as frequency and percentage for categorical variables. The distribution of continuous variables was determined by Kolmogorov–Smirnov test. Comparisons of quantitative variables between 2 groups were made by Mann–Whitney U test and Independent-samples t-test. Categorical data were compared by Chi-square test. Statistical significance was considered at *p* value < 0.05. The statistical analysis was performed in SPSS version 22.

## Results

### Demographic and clinical characteristics of patients

A total of 103 children with SJIA in Rheumatology Clinic database were eligible. Fifteen were excluded due to insufficient data (*n* = 14) and death (*n* = 1), while 49 refused to participate in the study. Most of these 49 patients were followed by telemedicine-based care owing to the Covid-19 pandemic. It was not possible for many patients to come to the hospital for performing the OGTT. Therefore, a total of 39 patients were enrolled in this study. Patient characteristics are shown in Table1. Their mean age (SD) was 12.0 (4.6) years. Twenty-one (54%) were male. Twenty-nine (74%) patients have entered puberty, their median Tanner stage was III. The median age (IQR) at diagnosis was 5.8 (3.7–9.3) years. Thirty-one out of 39 patients (80%) had inactive disease (status 3) and 7 (18%) had only arthritis (status 2). The remaining 1 patient had systemic features and arthritis (status 1). Median disease duration (IQR) was 65 (45–117) months. Median BMI SDS (IQR) was 0.1 (-0.5 to 1.7). Four patients (10%) were overweight (OW) and seven (18%) were obese (OB). Median (IQR) cumulative dose of prednisolone was 272 mg/kg (113–661). We reviewed baseline characteristics of 49 SJIA patients who refused to participate in the study due to inconvenience. Comparing between these 49 patients and the 39 patients who were enrolled, there were no significant differences in age, sex, BMI, BMI SDS, percentage of OW/OB, prednisolone use and disease status.

**Table 1:** Comparison of characteristics between lean and OW/OB patients with systemic juvenile idiopathic arthritis (SJIA)

Variables	Total (*n* = 39)	Lean (*n* = 28)	OW/OB (*n* = 11)	*p-*value
Age (years), mean (SD)	12.0 (4.6)	12.1 (5.2)	12 (3.1)	0.970
Gender, M (%)	21 (54)	14 (50)	7 (67)	0.442
Puberty, N (%)				0.693
Prepuberty (Tanner I)	10 (26)	8 (29)	2 (18)	
Puberty (Tanner II-V)	29 (74)	20 (71)	9 (82)	
BMI (kg/m^2^), mean (SD)	20.6 (5.9)	18.1 (3.4)	27.6 (5.4)	**0.001**
BMI SDS, median (IQR)	0.09 (-0.47 to 1.69)	-0.1 (-0.8 to -0.2)	2.3 (1.8–3.3)	**0.001**
Current prednisolone use, N (%) Dose (mg/kg/day), median (IQR)	13 (33) 0.5 (0.17–1.02)	7 (25) 0.23 (0.11–0.67)	6 (55) 0.63 (0.22–1.05)	0.131 0.199
Cumulative prednisolone dose (mg/kg), median (IQR)	272 (113–661)	260 (90–616)	272 (124–1,057)	0.473
Cumulative methotrexate dose (mg/kg), median (IQR)	1,913 (28–5,406)	1,566 (46–5,660)	2,565 (0–5,172)	0.826
History of biologics use, N (%)	17 (43)	11 (39)	6 (55)	0.482
Disease duration (months), median (IQR)	65 (45–117)	65 (46–110)	63 (25–132)	0.743
Systemic score, median (IQR)	0.4 (0.2–0.6)	0.4 (0.2–0.5)	0.4 (0.3–0.6)	0.492
Cumulative JADAS-27, median (IQR)	52 (33–83)	54 (31–85)	52 (45–71)	0.950
Disease status, N (%)				0.062
Status 1: systemic features	1 (2)	0 (0)	1 (9)	
Status 2: arthritis only	7 (18)	7 (25)	0 (0)	
Status 3: inactive disease	31 (80)	21 (75)	10 (91)	
Dyslipidemia, N (%)	22 (56)	14 (50)	8 (73)	0.288
TG (mg/dL), mean (SD)	96.8 (49.0)	88.8 (44.6)	116.9 (55.2)	0.107
TC (mg/dL), mean (SD)	177.9 (44.4)	174.4 (48.5)	187.0 (32.2)	0.431
HDL-C (mg/dL), mean (SD)	51.5 (12.1)	52.9 (12.4)	47.7 (11.2)	0.231
LDL-C (mg/dL), mean (SD)	116.4 (40.7)	112.7 (44.6)	125.9 (28.1)	0.368
Metabolic syndrome, N (%)	3 (7.7)	0 (0)	3 (27)	**0.018**
IGT, N (%)	1 (2.6)	0 (0)	1 (9)	0.282
FPG (mg/dL), mean (SD)	80.7 (6.4)	80.6 (5.3)	80.2 (8.7)	0.942
FPI (µIU/mL), median (IQR)	8.6 (4–13)	6 (3–10)	13 (11–15)	**0.003**
HOMA-IR, median (IQR)	1.8 (0.8–2.8)	1.2 (0.7–2.1)	2.6 (2.1–3.3)	**0.003**
WBISI, median (IQR)	5.0 (3.6–10.2)	5.8 (4.2–12.1)	3.7 (2.7–5.9)	**0.042**
IGI, median (IQR)	2.3 (1.3–3.4)	2.0 (0.9–3.4)	2.5 (2.0–3.5)	0.303
AUC-I / AUC-G, median (IQR)	0.6 (0.3–0.8)	0.3 (0.5–0.8)	0.7 (0.5–1.0)	0.254
DI, median (IQR)	13.6 (6.8–24.3)	14.1 (8.9–25.2)	8.6 (5.3–20.4)	0.454

*AUC-G* area under the curve of glucose (mg⋅min/dL), *AUC-I* area under the curve of insulin (µIU⋅min/mL), *AUC-I / AUC-G* (µIU⋅dL/mg⋅mL), *BMI* body mass index, *DI* disposition index (dL^2^/mg⋅mL), *FPG* fasting plasma glucose, *FPI* fasting plasma insulin, *HDL-C* high density lipoprotein cholesterol, *HOMA-IR* homeostasis model assessment for insulin resistance (µIU⋅mg/mg⋅dL), *IGI* insulinogenic index (µIU⋅dL/mg⋅mL), *IGT* impaired glucose tolerance, *IQR* interquartile range, *JADAS* Juvenile Arthritis Disease Activity Score, *LDL-C* low density lipoprotein cholesterol, M male, *OW/OB* overweight/obesity, *SD* standard deviation, *TC* total cholesterol, *TG* triglyceride, *WBISI* whole-body insulin sensitivity index (mL⋅dL/µIU⋅mg)

Only 1 obese patient had prediabetes and none had diabetes. Three patients had hypertension and fulfilled criteria of metabolic syndrome. Twenty-two patients (56%) had dyslipidemia including decreased HDL-C (*n* = 7, 18%), elevated TG (*n* = 12, 31%), elevated low density lipoprotein-cholesterol (LDL-C) (*n* = 12, 31%) and elevated both TG and LDL-C (*n* = 5, 13%).

The patient with prediabetes was a 15-year old Thai male with severe obesity (BMI 39.2 kg/m^2^, BMI SDS 3.6). He was diagnosed with SJIA at the age of 11 years with macrophage activation syndrome, obstructive sleep apnea and hypertriglyceridemia (TG = 259 mg/dL). His disease was in remission at age 14 years. He had discontinued all medications for one year prior to the study. He also had hypertriglyceridemia (TG = 129 mg/dL) and reduced HDL-C (34 mg/dL). He had no family history of diabetes.

### Beta cell function and insulin sensitivity in patients with SJIA

Patients with SJIA were divided into 2 groups: lean (*n* = 28) and OW/OB (*n* = 11). Sex, age, Tanner stage, disease duration, systemic score, JADAS-27, disease status, cumulative dose of prednisolone, MTX, NSAIDs, biologic use and treatment duration were not significantly different between the 2 groups. In comparison with the lean group, current prednisolone use and cumulative prednisolone dose tended to be higher in the OW/OB group but were not significantly different. Three of 11 (27%) OW/OB patients had metabolic syndrome, and 1 of 11 (9%) had impaired glucose tolerance. In comparison with lean patients, OW/OB had a significantly higher fasting plasma insulin level and HOMA-IR but significantly lower WBISI, while no difference in fasting plasma glucose level (Table 1).

**Fig. 1:**
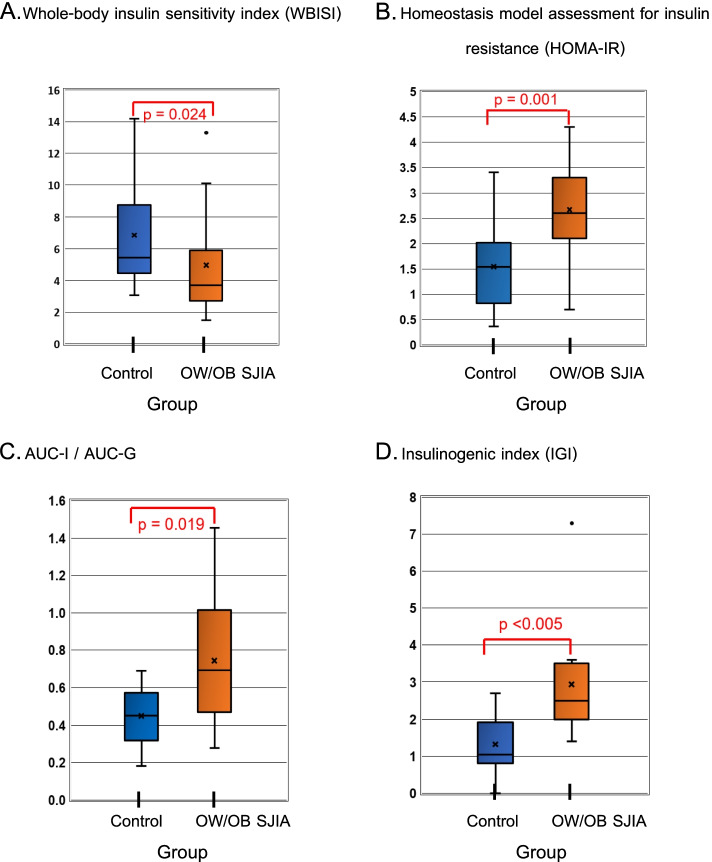
Comparison of insulin sensitivity and insulin secretion indices between OW/OB controls (*n* = 33) and OW/OB patients with systemic juvenile idiopathic arthritis (SJIA) (*n* = 11). Insulin sensitivity indices shown in **A** and **B**; insulin secretion indices shown in **C** and **D**; data expressed as median (interquartile range); **A**, Whole-body insulin sensitivity index (WBISI) (mL⋅dL/µIU⋅mg); **B**, Homeostasis model assessment for insulin resistance (HOMA-IR) (µIU⋅mg/mg⋅dL); **C**, Area under the curve (AUC) of insulin to AUC of glucose ratio (µIU⋅dL/mg⋅mL) during OGTT; **D**, Insulinogenic index (IGI) (µIU⋅dL/mg⋅mL)

Due to the fact that obesity affects glucose metabolism, OW/OB patients (*n* = 11) were compared with sex- puberty- and age-matched OW/OB controls (*n* = 33). Of 11 OW/OB patients, 10 had disease status 3 (inactive) and only 1 had active disease (status 1). Six patients were currently treated with low dose prednisolone. The controls were recruited from healthy OW/OB children who had OGTT performed in Pediatric Endocrinology Clinic. In comparison with the OW/OB controls, OW/OB patients with SJIA (*n* = 11) had higher insulin resistance index, as shown by median (IQR) homeostasis model assessment for insulin resistance (HOMA-IR) [2.6 (2.1–3.3) *vs* 1.5 (0.8–2.0), *p* = *0.001*], and lower insulin sensitivity index as shown by median (IQR) whole-body insulin sensitivity index (WBISI) [3.7 (2.7–5.9) *vs* 5.4 (4.5–8.7), *p* = *0.024*]. The former had compensatory increased β-cell function indices as compared to the latter [median (IQR) insulinogenic index (IGI), 2.5 (2.0–3.5) *vs* 1.0 (0.8–1.9), *p* < *0.005*; median (IQR) AUC-I/AUC-G, 0.7 (0.5–1.0) *vs* 0.4 (0.3–0.6), *p* = 0.019] (Table 2 and Fig. 1).

**Table 2:** Comparison of characteristics and insulin sensitivity indices between OW/OB controls and OW/OB patients with systemic juvenile idiopathic arthritis (SJIA)

Variables	Control (*n* = 33)	OW/OB SJIA (*n* = 11)	*p*-value
Age (years), mean (SD)	13 (3.4)	12 (3.1)	0.492
Gender, M (%)	19 (58)	7 (64)	1.000
Puberty, N (%)			0.701
Prepuberty (Tanner I)	9 (27)	2 (18)	
Puberty (Tanner II-V)	24 (73)	9 (82)	
BMI (kg/m^2^), mean (SD)	28.4 (4.5)	27.6 (5.1)	0.664
BMI SDS, median (IQR)	2.4 (2.0–2.7)	2.3 (1.9–3.3)	0.978
FPG (mg/dL), mean (SD)	85 (6.8)	81 (8.8)	0.296
FPI (µIU/mL), median (IQR)	7.5 (3.9–10.3)	13 (11–15)	**0.001**
HOMA-IR, median (IQR)	1.5 (0.8–2.0)	2.6 (2.1–3.3)	**0.001**
WBISI, median (IQR)	5.4 (4.5–8.7)	3.7 (2.7–5.9)	**0.024**
IGI, median (IQR)	1.0 (0.8–1.9)	2.5 (2.0–3.5)	**0.001**
AUC-I / AUC-G, median (IQR)	0.4 (0.3–0.6)	0.7 (0.5–1.0)	**0.019**
DI, median (IQR)	7.5 (4.8–11.1)	8.6 (5.3–20.4)	0.247

*AUC-G* area under the curve of glucose (mg⋅min/dL), *AUC-I* area under the curve of insulin (µIU⋅min/mL), *AUC-I / AUC-G* (µIU⋅dL/mg⋅mL), *BMI* body mass index, *DI* disposition index (dL^2^/mg⋅mL), *FPG* fasting plasma glucose, *FPI* fasting plasma insulin, *HOMA-IR* homeostasis model assessment for insulin resistance (µIU⋅mg/mg⋅dL), *IGI* insulinogenic index (µIU⋅dL/mg⋅mL), *IQR* interquartile range, *M* male, *OW/OB* overweight/obesity, *SD* standard deviation, *WBISI* whole-body insulin sensitivity index (mL⋅dL/µIU⋅mg)

Since glucocorticoid treatment affects glucose metabolism, OW/OB patients currently treated with low dose prednisolone (2.5–10 mg/day) (*n* = 6) were compared with those without prednisolone treatment (*n* = 5). There were no significant differences in insulin sensitivity indices and β-cell function indices (data not shown). In addition, concerning cumulative prednisolone dose, comparing between patients who received the cumulative prednisolone doses of < 250 mg/kg (*n* = 5) and > 250 mg/kg (*n* = 6), there were no differences in all insulin sensitivity and β-cell function indices (data not shown).

Due to unavailable to recruit healthy lean controls to have OGTT, we compared our lean patients (*n* = 28) with the previously published normal weight children (*n* = 142) [25]. Fasting plasma glucose, insulin resistance index (HOMA-IR) and fasting plasma insulin in the patients seem to be comparable to those of lean controls (Table 3).

**Table 3:** Comparison of characteristics and insulin sensitivity indices between lean controls and lean patients with systemic juvenile idiopathic arthritis (SJIA)

	Control (*n* = 142)*			Lean SJIA (*n* = 28)		
	**Mean (SD)**	**Median**	**Range**	**Mean (SD)**	**Median**	**Range**
Age (years)	10.6 (3.8)	10.8	3–19	12.1 (5.2)	12.5	4–25
BMI (kg/m^2^)	18 (2.4)	17.0	14.2–24.5	18 (3.4)	16.7	13.2–24.7
BMI SDS	-0.35 (0.08)	-0.42	-1.94 to 1.74	-0.35 (0.90)	-0.07	-2.72 to 0.78
FPG (mg/dL)	84 (5.8)	85	64–100	81 (5.4)	80	70–95
FPI (µIU/mL)	7 (4.3)	6.15	1–23	6 (4.9)	6.05	1–19
HOMA-IR	1.5 (0.9)	1.3	0.2–5.4	1.5 (1.0)	1.2	0.2–4.2

^*^Reference number [24]

*BMI* body mass index, *FPG* fasting plasma glucose, *FPI* fasting plasma insulin, *HOMA-IR* homeostasis model assessment for insulin resistance (µIU⋅mg/mg⋅dL), *NA* not available *SD* standard deviation

Since chronic inflammation causes insulin resistance, lean patients with SJIA (*n* = 28) in disease status 2 (arthritis only) (*n* = 7) were compared with those in status 3 (inactive disease) (*n* = 21). There were no significant differences in insulin sensitivity and insulin secretion indices (data not shown). Concerning prednisolone effect on these indices in the 28 lean SJIA patients, 7 (25%) were treated with low dose prednisolone at the time of the study enrollment. Comparing between these 7 patients and 21 patients who had no prednisolone treatment at the time of study enrollment, there were no differences in all insulin sensitivity and β-cell function indices.

## Discussion

This study is a cross-sectional evaluation of glucose metabolism in children with SJIA. Of 39 patients with SJIA, only one had impaired glucose tolerance, none had diabetes. In comparison with age- and sex- matched healthy OW/OB children, OW/OB patients with SJIA had lower insulin sensitivity indices and higher insulin secretion indices consistent with compensatory increased β-cell function.

In adults with rheumatoid arthritis (RA), many previous studies have demonstrated that there was an increased risk for T2DM [6]. Prevalence rate of T2DM in adults with RA was about 15%-19%, which was significantly higher than the prevalence rate of 4%-8% of global middle-aged population [5]. However, diabetes was not found among children with SJIA in this study. In healthy severely obese children, it usually takes many years to develop abnormal glucose metabolism [26]. Although chronic inflammation causes insulin resistance, and consequently development of prediabetes and diabetes [8], young children with SJIA may not yet develop abnormal glucose metabolism. In addition, since most patients had inactive disease at the time of study, inflammation-induced insulin resistance, if any, could be lessened. However, in comparison with healthy OW/OB children, OW/OB children with SJIA in this study had higher insulin resistance index [HOMA-IR] and lower insulin sensitivity index [WBISI] which suggest that the patients could have an increased risk for developing diabetes and may develop diabetes in the future.

In lean patients with SJIA in this study, insulin sensitivity indices seemed to be comparable with those of lean controls in the previous report [25]. Therefore, evidences of insulin resistance in lean patients with SJIA may not yet appear in the young. Long-term follow up is required to demonstrate abnormal glucose metabolism, if any, in the future.

This preliminary study reported increased insulin resistance in young OW/OB children with SJIA. To our knowledge, this finding has not been reported. Nevertheless, there are several limitations in this study. First, less than half of the patients could be enrolled and the obese control group was not a case–control in design. These could cause selection biases. However, those patients who refused to participate in this study due to the Covid-19 pandemic, their baseline characteristics were not different from the studied group. Second, small sample size led to only one index patient with prediabetes found and most of the patients studied were inactive cases. In fact, most patients in our Rheumatology Clinic were inactive cases. In addition, during severe active disease, critically unwell patients who received high-dose corticosteroids and did not have adequate dietary intake, were not appropriate to perform the OGTT. However, mildly active and inactive cases may still have low grade inflammation. In addition, the control group was obtained from a hospital setting rather than being community controls so they may not truly representation of the non-SJIA population. Third, prediabetes and diabetes in young patients with SJIA particularly those without obesity may be rare or may not yet occur. Fourth, current unavoidable glucocorticoid treatment, albeit relatively low dose, could affect insulin sensitivity and glucose metabolism, and at least in part leads to increased insulin resistance and impaired glucose tolerance. Obviously, the more severity and chronicity of SJIA, the more prolonged glucocorticoid treatment, and the more severe degree of obesity, the higher risks for developing prediabetes and diabetes could be apparent.

## Conclusions

SJIA children with obesity seemed to have increased insulin resistance as compared to healthy obese children. Thus, long term vigilance for development of prediabetes/diabetes in children with SJIA may be essential. Longitudinal studies on glucose metabolism in children with SJIA are required.

## Data Availability

The datasets generated and analyzed during the current study are not publicly available because the sharing could compromise individual privacy. Data are available from the corresponding author upon reasonable request.

## Abbreviations

AUC-G: Area under the curve of glucose
AUC-I: Area under the curve of insulin
BMI: Body mass index
DI: Disposition index
DMARD: Disease-modifying anti-rheumatic drugs
FPG: Fasting plasma glucose
FPI: Fasting plasma insulin
HDL-C: High density lipoprotein cholesterol
HOMA-IR: Homeostasis model assessment for insulin resistance
IFG: Impaired fasting glucose
IGI: Insulinogenic index
IGT: Impaired glucose tolerance
IQR: Interquartile range
JADAS: Juvenile Arthritis Disease Activity Score
LDL-C: Low density lipoprotein cholesterol
M: Male
MS: Metabolic syndrome
MTX: Methotrexate
NA: Not available
NSAIDs: Nonsteroidal anti-inflammatory drugs
OB: Obese
OGTT: Oral glucose tolerance test
OW: Overweight
RA: Rheumatoid arthritis
SD: Standard deviation
SJIA: Systemic juvenile idiopathic arthritis
T2DM: Type 2 diabetes mellitus
TC: Total cholesterol
TG: Triglyceride
WBISI: Whole-body insulin sensitivity index

## Declarations

### Completing interests

The authors declare that there are no completing interests.
